# Influence of age and sex on hospitalization of nursing home residents: A cross-sectional study from Germany

**DOI:** 10.1186/s12913-017-2008-7

**Published:** 2017-01-19

**Authors:** Falk Hoffmann, Guido Schmiemann

**Affiliations:** 1 Department of Health Services Research, Carl von Ossietzky University Oldenburg, Ammerländer Heerstr. 140, D- 26111 Oldenburg, Germany; 2 Institute for Public Health and Nursing Science, Department for Health Services Research, University of Bremen, Bremen, Germany; 3 Health Sciences Bremen, University of Bremen, Bremen, Germany

**Keywords:** Nursing homes, Health services research, Epidemiology, Care dependency, Germany

## Abstract

**Background:**

Nursing homes residents (NHR) are frequently transferred to hospitals. There is some evidence that male NHR are more often hospitalized than females, but the influence of age is less clear and predictors might differ between sexes. Analyses according to age groups between males and females have only been investigated once and none of the existing studies have conducted multivariate analyses stratified by sex. Aim of this study was to fill this gap.

**Methods:**

We used data of the “Inappropriate Medication in patients with REnal insufficiency in Nursing homes” (IMREN) study, which was conducted between October 2014 and April 2015 in nursing homes in northwestern Germany (Bremen and Lower Saxony). Anonymised data was obtained by nursing staff of the participating nursing homes. All residents living in the participating care units were included. We assessed whether they were hospitalized at least once during the preceding 12 months. Cluster-adjusted multivariate logistic regression was applied to identify variables associated with hospitalizations. All analyses were stratified by sex.

**Results:**

Of 852 residents from 21 nursing homes (mean age 83.5 years; 76.5% females), 43.1% (95% confidence intervals [95% CI]: 35.6–50.5) were hospitalized at least once during the preceding 12 months. This proportion was higher in residents institutionalized within the last 6 months compared to those with a longer length of stay (65.7% vs. 39.5%). Although not statistically significant, males were more often hospitalized than females (52.4% vs. 40.3%) and differences between sexes are particularly remarkable for age, health status and length of stay. In females, the chance of being hospitalized decreased steadily with age (OR: 2.40 [95% CI: 1.24–4.64] and 1.60 [95% CI: 1.05–2.43] for age groups <75 and 75–84 years compared to 85+ years). On the other hand, males aged 75–84 years had a statistically significant lower chance compared to 85+ years olds (OR: 0.41; 95% CI: 0.19–0.90).

**Conclusions:**

Differences in factors associated with hospitalizations might exist between sexes. We strongly suggest that further studies on hospitalizations of NHR should stratify their analyses by sex.

## Background

The worldwide epidemic of chronic non-communicable diseases, which is strongly linked to population ageing, also leads to a substantial increase in the number of older care-dependent persons [1]. In Germany, about 30% of older care-dependent persons are living in nursing homes and the total numbers are increasing over recent years [2].

Residents of nursing homes are frequently transferred to hospitals, especially shortly after admission and near to death [3, 4]. Such hospitalizations might have negative clinical consequences [5, 6], and a large proportion of them are deemed to be avoidable [7–9]. A review of the literature revealed that hospitalization rates of nursing home residents vary widely between 9 and 59% across different geographic areas, types of nursing homes, populations and time periods [3]. The most consistent finding is that male residents are more often hospitalized than females [3, 10–14]. On the other hand, the influence of age is less clear. Although it has been shown that age is positively associated with hospitalizations of nursing home residents [3], some studies found decreasing rates of hospitalization above the age of 80–85 years [10–15].

Such findings might be influenced by differences between sexes, because females make up a larger proportion of residents, are older and generally have a higher disease burden than males [16, 17]. However, analyses on hospitalizations of nursing home residents are usually not presented stratified by sex and such differences do not receive much attention in the literature. In a recently published systematic review, we found that 20 studies assessed the influence of sex and all found that hospitalizations are more often in male residents [18]. However, just 4 of these studies discussed these findings at all [10, 11, 19, 20], only one presented analyses according to age groups between males and females [10] and none of the existing studies have conducted multivariate analyses stratified by sex [18]. Therefore, it is also not clear whether differences are present in male and female residents with comparable health status or if predictors of hospitalization differ between sexes. Such differences between sexes do not receive much attention in research on nursing home residents, although it has been shown that patterns of care needs, chronic medical conditions and service utilization differ between male and female residents [16, 17]. However, understanding sex-dependent differences is essential to identify oversupply or undersupply of health services and to optimise care.

Therefore, the aim of this study was to investigate the proportion of nursing home residents in Germany being hospitalized by age and health status as well as to assess factors associated with hospitalization, both stratified by sex.

## Methods

### Design, study population and variables

Data of the “Inappropriate Medication in patients with REnal insufficiency in Nursing homes” (IMREN) study were used. IMREN is a cross-sectional study that was conducted between October 2014 and April 2015 in nursing homes in Bremen and Lower Saxony. We recruited a convenience sample of nursing homes that were heterogeneous in terms of urban and rural location, size and supporting organization. The primary aim of the IMREN study was to determine the prevalence of renal insufficiency in nursing home residents, for which a total sample size of 856 residents in 19 care units was estimated. Further analyses have been published elsewhere [21–23].

Nursing homes were required to include all residents living in the participating care units. No exclusion criteria existed. Anonymised data was obtained by nursing staff of the participating nursing homes using a piloted questionnaire to which the current medication plan was appended. Because only data already available were obtained, active participation and informed consent of the residents were not required. For each resident, several socio-demographic and health related data were collected.

Age, sex, length of residence, selected chronic diseases and care dependency were assessed from the residents’ records. Care dependency was evaluated in terms of care levels determined by the compulsory Long-Term Care Insurance. Benefits in Long-Term Care Insurance are only available upon application to those who require support in their activities of daily living including personal hygiene, eating and mobility. The Medical Review Board evaluates the applicants and classifies them into one of the three levels of care dependency corresponding to the estimated time required for assistance ranging from care level I (considerable need of care) to level III (most heavily care dependent). Further information on the German long-term care system can be found in Busse and Blümel [24] as well as Rothgang [25]. Whether residents were hospitalized at least once during the preceding 12 months was also determined from the residents’ records.

Besides the levels of care dependency indicating physical disabilities we also used the number of prescribed medications as a measure of health status like in a recent paper [26]. It has been shown that the number of distinct medications prescribed performed well as a predictor for health services utilization and mortality in older persons [27]. For our analyses, we categorised the number of scheduled medications (excluding drugs that were prescribed as-needed only) into quartiles.

The study complies with the Declaration of Helsinki and was approved by the ethics committee of the University of Bremen.

### Statistical analysis

All analyses were stratified by sex. After a descriptive characterisation of the study cohort, the proportion of residents that were hospitalized during the preceding 12 months was estimated alongside with 95% confidence intervals (95% CI) adjusting for cluster sampling. These analyses were also stratified by age (<75; 75–84 and 85+ years), level of care dependency (3 categories), quartiles of the numbers of scheduled medications (4 categories) and length of residence (2 categories).

Furthermore, multivariate logistic regression was applied to identify which variables were associated with at least one hospitalization during the preceding 12 months. The above mentioned variables age, level of care dependency, quartiles of the numbers of scheduled medications and length of residence were included in the model using the same categories. The regression was also stratified by sex. Analyses were cluster-adjusted using mixed models with random effects.

In order to estimate required sample sizes in future cluster-based studies in nursing homes, the intra-cluster correlation coefficient (ICC) for hospitalizations was also calculated [28].

We performed all statistical analyses with SAS for Windows version 9.4 (SAS Institute Inc., Cary, NC).

## Results

### Baseline characteristics

A total of 852 residents from 21 nursing homes were included (ranging between 10 and 69 per facility). They were on average 83.5 years old and more than three quarters were females (Table 1). Nearly all residents (98.3%) were assigned to one of the three care levels with one quarter having care level III. The most common chronic diseases were hypertension (59.3%) and dementia (57.7%). The average number of scheduled medications was 6.3 per resident.

**Table 1:** Baseline characteristics of nursing home residents, by sex

Baseline characteristics	Males (*n* = 199; 23.5%)	Females (*n* = 647; 76.5%)	Total (*n* = 852^a^; 100%)
Age in years, Mean ± SD	78.3 ± 11.4	85.0 ± 9.7	83.5 ± 10.5
Age groups, in years			
<75	29.6%	9.4%	14.1%
75-84	36.2%	26.9%	29.1%
85+	34.2%	63.7%	56.8%
Length of residence in years, Mean ± SD	2.8 ± 3.5	3.3 ± 3.3	3.2 ± 3.4
Legal guardian designated	53.7%	44.1%	46.2%
Level of care dependency			
None/I	40.2%	40.1%	40.3%
II	33.5%	34.9%	34.3%
III	26.3%	25.0%	25.4%
Indwelling urinary catheter	25.3%	9.7%	13.4%
Feeding tube	6.6%	3.4%	4.1%
Chronic diagnoses			
Hypertension	59.9%	59.0%	59.3%
Dementia	51.0%	59.5%	57.7%
Heart failure	19.4%	23.5%	22.5%
Diabetes	25.3%	20.9%	21.9%
Stroke	26.5%	19.4%	21.3%
Number of scheduled medications, Mean ± SD	6.2 ± 3.1	6.3 ± 3.4	6.3 ± 3.4

^a^Numbers may vary due to missing values, ranging between 0 (age) and 34 (legal guardian designated)

Baseline characteristics stratified by sex are also displayed in Table 1. Males were younger than females and had more often an indwelling urinary catheter or feeding tube. On the other hand, there were no differences between the distribution of care levels and the number of medications taken between sexes. Females were more often suffering from dementia but had less often a history of previous stroke or diabetes compared to males.

### Hospitalization

A total of 43.1% (95% CI: 35.6–50.5) were hospitalized at least once during the preceding 12 months (Table 2). The intra-cluster correlation coefficient (ICC) for hospitalizations was estimated to be 0.0789.

**Table 2:** Hospitalization of nursing home residents during the preceding 12 months (with 95% CI), by sex

Characteristic	Males (*n* = 191)	Females (*n* = 613)	Total (*n* = 810^a^)
Age groups, in years			
< 75	61.4% (49.3–73.6)	52.6% (35.9–69.4)	57.0% (46.4–67.7)
75–84	38.6% (27.7–49.4)	46.1% (34.3–57.8)	43.9% (34.9–52.8)
85+	59.4% (45.6–73.1)	36.1% (26.1–46.0)	39.2% (29.8–48.6)
Level of care dependency			
None/I	55.3% (44.1–66.4)	36.7% (27.0–46.3)	41.3% (32.7–49.8)
II	56.5% (45.8–67.1)	40.6% (30.2–51.0)	44.2% (34.9–53.4)
III	42.9% (29.5–56.3)	44.8% (32.9–56.7)	43.9% (34.7–53.1)
Number of scheduled medications			
Q1 (0–4)	52.6% (35.9–69.4)	32.1% (21.0–43.2)	36.9% (26.0–47.9)
Q2 (5–6)	39.5% (23.3–55.8)	36.8% (27.1–46.5)	37.2% (28.0–46.4)
Q3 (7–9)	57.8% (45.5–70.1)	45.9% (35.7–56.2)	48.6% (40.3–56.9)
Q4 (10+)	59.3% (46.4–72.1)	49.1% (36.3–61.9)	51.4% (41.1–61.8)
Length of residence			
≤ 180 days	81.6% (64.3–98.8)	55.9% (40.1–71.6)	65.7% (52.2–79.3)
181+ days	44.7% (37.1–52.4)	38.3% (29.8–46.8)	39.5% (32.0–47.0)
Total	52.4% (45.8–58.9)	40.3% (31.9–48.7)	43.1% (35.6–50.5)

^a^Numbers may vary due to missing values, ranging between 0 (age) and 11 (level of care dependency)

Although not statistically significant, males were more often hospitalized than females (52.4% vs. 40.3%). These differences were also found when persons with chronic diagnoses (hypertension, dementia, heart failure, diabetes and stroke) were analyzed and diabetes was the only condition for which no difference in the proportion being hospitalized between males and females was seen (data not shown). Differences were also found in some of the stratified analyses according to the degree of care dependency and the number of prescribed medications, which were used as measures of health status. They are especially remarkable in residents with a lower degree of morbidity. Male residents with a low level of care dependency are much more often hospitalized than females (no level of care dependency or level I: 55.3% vs. 36.7%). The same holds true for those receiving 0–4 scheduled medications (52.6% vs. 32.1%).

Further differences between sexes were found according to age. In females, the proportion hospitalized at least once during the preceding 12 months decreased steadily with increasing age (52.6; 46.1 and 36.1% for age groups <75; 75–84 and 85+ years), whereas this trend was not found in males (61.4; 38.6 and 59.4%, respectively).

Residents institutionalized within the last 6 months (13.8% of our cohort) were more often hospitalized during the preceding 12 months than those with a longer length of residence (65.7% vs. 39.5%). Again, we found differences between sexes that were pronounced in those with a shorter length of stay.

### Multivariate regression

Results of the multivariate analyses are shown in Table 3. In the overall model, there was a numerically but not statistically significant increased chance for males of being hospitalized. Age <75 years as well as length of residence of no longer than 180 days was associated with hospitalizations. The chance for being hospitalized also increased with a higher number of medications.

**Table 3:** Multivariate logistic regression on factors associated with hospitalization of nursing home residents during the preceding 12 months (OR with 95% CI), by sex

Characteristic	Males (*n* = 186)	Females (*n* = 599)	Total (*n* = 785)
Sex			
Female			1
Male			1.35 (0.92–1.98)
Age groups, in years			
< 75	1.30 (0.57–2.96)	2.40 (1.24–4.64)	2.14 (1.30–3.53)
75–84	0.41 (0.19–0.90)	1.60 (1.05–2.43)	1.12 (0.78–1.61)
85+	1	1	1
Level of care dependency			
None/I	1	1	1
II	1.09 (0.51–2.31)	1.21 (0.79–1.85)	1.18 (0.82–1.69)
III	0.62 (0.27–1.42)	1.68 (1.03–2.75)	1.28 (0.85–1.93)
Number of scheduled medications			
Q1 (0–4)	1	1	1
Q2 (5–6)	0.50 (0.20–1.23)	1.28 (0.75–2.16)	1.05 (0.68–1.64)
Q3 (7–9)	1.38 (0.62–3.07)	2.47 (1.52–4.03)	2.05 (1.36–3.07)
Q4 (10+)	1.40 (0.50–3.93)	2.78 (1.58–4.91)	2.43 (1.49–3.95)
Length of residence			
≤ 180 days	4.99 (1.98–12.59)	2.43 (1.36–4.35)	2.94 (1.82–4.74)
181+ days	1	1	1

However, results of the overall model are largely influenced by the fact, that more than three quarters of residents were females. When stratifying the analyses by sex, there are large differences (Table 3). In females, the chance of being hospitalized decreased steadily with age (OR: 2.40 and 1.60 for age groups <75 and 75–84 years compared to 85+ years). On the other hand, males aged 75–84 years had a statistically significant lower chance compared to 85+ years olds (OR: 0.41). We also found an interaction between sex and age groups in the unstratified model (*p* = 0.0071).

Furthermore, a higher degree of care dependency and a higher number of prescribed medications were associated with an increased chance of hospitalizations only in females. In males, this was not the case and not even a numerical increase or trend was found. The influence of being institutionalized within the last 6 months on hospitalizations was higher in males than in females (OR: 4.99 and 2.43). However, there were no further significant interaction effects between sex and other variables besides age groups.

## Discussion

### Findings, comparison with other studies and interpretation

To our knowledge, this is one of the first studies comparing hospitalizations of nursing home residents and associated factors by sex. We found that male nursing home residents were 1.35-fold more often hospitalized during the preceding 12 months than females and that there seem to be differences in factors associated with hospitalizations between males and females. The chance of being hospitalized increased with decreasing age, increasing care dependency as well as increasing number of medications only in females. In males, these clear trends were not found and there were differences between sexes on the influence of length of stay and hospitalizations.

The literature indicates that hospitalizations of nursing home residents are relatively common but estimates vary widely across studies [3, 18]. Besides different geographic areas and types of nursing homes or hospitalizations, the published studies use a wide range of measures, time periods and populations, making comparisons difficult. Hospitalizations are highest for persons before and shortly after being newly admitted to nursing homes and decline with length of stay [3, 11, 29, 30]. This was also observed in our study, where persons institutionalized within the last 6 months were more often hospitalized during the preceding 12 months than residents with a longer length of stay. When compared to studies also including prevalent nursing home residents [3, 14, 18, 31, 32], the proportion with one or more hospitalizations is much higher in our study than those reported from other western countries. These comparably high hospitalization rates in Germany have already been pointed out by Ramroth et al. [11] in an earlier study using data that are now 15 years old.

Although not statistically significant in our study, the finding that male nursing home residents are more often hospitalized than females is consistent with the existing literature [3, 10–14, 18]. However, in our systematic review [18] we only found one study that reported further analyses stratified by sex and age [10] and no study systematically assessed reasons for these differences or compared predictors of hospitalizations in multivariate analyses between sexes. However, understanding such sex-dependent patterns is a prerequisite to assess whether oversupply or undersupply might exist and to optimise care, but data on care needs, chronic medical conditions and service utilization in nursing home residents are often presented in aggregate for both sexes [16].

Our most striking finding was that age had a quite different influence between sexes. Ronald et al., as the only study we found that presented hospitalization rates by age groups and sex, also revealed some differences [10]. In females, they found a decrease in hospitalizations with increasing age, which is in line with our findings. Their results show only small differences between age groups in males. Although many studies adjust for sex in multivariate analyses [14, 18, 29, 33], these results are largely influenced by the fact that about three quarters of nursing home residents are females. While it seems to be established that healthier nursing home residents are less frequently admitted to hospitals [3, 33], we also found striking sex differences and this association was only seen in female residents. We used the level of care dependency and the number of scheduled medication as proxies for health status. For the quartiles of scheduled medications, which might be a better proxy for morbidity, we also found a clear trend that an increased number goes in line with an increased proportion of residents being hospitalized in females. When studying levels of care dependency, which reflect physical disabilities, this trend was weaker but, again, only shown in females. This phenomenon should be studied further in order to understand sex differences in hospitalizations of nursing home residents.

### Strengths and limitations

Major strength of our study was that we were able to collect data of all residents living in the participating care units including their medication plans. Data were captured by the respective nursing staff and many health related information could be obtained from the residents’ records. However, some limitations have to be considered. First, we only included nursing homes in the northwestern part of Germany that were willing to participate, which might result in a selection bias and may impact the external validity of our findings. However, we tried to consider a heterogeneous sample of nursing homes including different sizes, providers and rural as well as urban regions. Second, although nursing staff should assess information on hospitalization from the residents’ records, a recall bias might be a further limitation in residents which were newly institutionalized or for which no data were available for other reasons. Besides whether residents were hospitalized at least once during the preceding 12 months, no further data on hospitalizations were assessed. Therefore, we do not have information on the number of hospitalizations, discharge diagnoses as well as length of hospital stay because we aimed to keep the burden of documentation as low as possible. Furthermore, for about one quarter (26.1%) length of stay was less than 12 months and some of their hospitalisations might have occurred before nursing home admission. Although there were missing values, data on hospitalization were missing for less than 5% of residents, which was quite small. We used a cross-sectional design and it was not possible to assess whether chronic conditions or changes in health status occur after hospital stay. However, our main objective was to investigate age and sex differences and these factors are not affected by hospitalization. Finally, our study is hampered by the small sample size, especially for males. The large confidence intervals indicate less accurate estimates and our results must be interpreted with some caution. However, especially the different trends between sexes are unlikely to be due to chance.

## Conclusions

We found that more than 4 out of 10 nursing home residents in Germany were hospitalized at least once during the preceding 12 months, which is quite higher when compared to studies from other western countries. Although not statistically significant, males were more often hospitalized than females. This finding is in line with the literature. Differences in associated factors between sexes are particularly remarkable for age, length of stay as well as for health status. Our findings should be confirmed in a larger sample. However, virtually no study assessed sex differences yet. We put up for discussion whether widely accepted and established factors associated with hospitalizations of nursing home residents have the same influence in males and females. Therefore, we strongly suggest that any further studies on hospitalizations of nursing home residents should stratify all of their analyses by sex. Also, studies should investigate differences between males and females in decisions and reasons for hospitalization, with a special regard on age, length of stay, the degree of morbidity and physical disabilities. Further studies are a prerequisite to understand whether these differences are due to different care needs between sexes or if there might be an oversupply in males or an undersupply in females.

## Abbreviations

95% CI: 95% confidence intervals
IMREN: Inappropriate Medication in patients with REnal insufficiency in Nursing homes
OR: Odds ratio
SD: Standard deviation
